# Molecular Detection and Characterization of Porcine Epidemic Diarrhea Virus and Porcine Aichivirus C Coinfection in México

**DOI:** 10.3390/v13050738

**Published:** 2021-04-23

**Authors:** Montserrat-Elemi García-Hernández, María-Elena Trujillo-Ortega, Sofía-Lizbeth Alcaraz-Estrada, Luis Lozano-Aguirre-Beltrán, Carlos Sandoval-Jaime, Blanca Itzel Taboada-Ramírez, Rosa-Elena Sarmiento-Silva

**Affiliations:** 1 Departamento de Microbiología e Inmunología, Facultad de Medicina Veterinaria y Zootecnia, Universidad Nacional Autónoma de México, Ciudad Universitaria, Av. Universidad #3000, Ciudad de México 04510, Mexico; elemi.gh@gmail.com; 2 Departamento de Medicina y Zootecnia de Cerdos, Facultad de Medicina Veterinaria y Zootecnia, Universidad Nacional Autónoma de México, Ciudad Universitaria, Av. Universidad #3000, Ciudad de México 04510, Mexico; elenam@unam.mx; 3 División de Medicina Genómica y Genética Clínica, Centro Médico Nacional “20 de Noviembre”, Instituto de Seguridad y Servicios Sociales de los Trabajadores del Estado, Av. Félix Cuevas #540, Ciudad de México 03100, Mexico; sofia.alcaraz@issste.gob.mx; 4 Centro de Ciencias Genómicas, Universidad Nacional Autónoma de México, Av. Universidad 2001, Cuernavaca 62209, Mexico; llozano@ccg.unam.mx; 5 Instituto de Biotecnología, Universidad Nacional Autónoma de México, Av. Universidad 2001, Cuernavaca 62209, Mexico; carlossj@ibt.unam.mx (C.S.-J.); btaboada@ibt.unam.mx (B.I.T.-R.)

**Keywords:** porcine diarrhea, kobuvirus, coinfection

## Abstract

Swine enteric viral infections are responsible for substantial economic losses in the pork industry worldwide. Porcine epidemic diarrhea (PEDV) is one of the main causative agents of diarrhea in lactating pigs, and reports of PEDV coinfection with other enteric viruses highlight the importance of viral interactions for disease presentation and outcomes. Using next-generation sequencing (NGS) and sequence analyses from samples taken from piglets with acute diarrhea, we explored the possible interactions between PEDV and other less reported pathogens. PEDV coinfection with porcine kobuvirus (PKV) was detected in 36.4% (27/74) of samples. Full genomes from porcine coronavirus and kobuvirus were obtained, as was a partial porcine sapovirus genome (PSaV). The phylogenetic results show the clustering of these strains corresponding to the geographical relationship. To our knowledge, this is the first full genome and isolation report for porcine kobuvirus in México, as well as the first phylogenetic analysis for porcine sapovirus in the country. The NGS approach provides a better perspective of circulating viruses and other pathogens in affected production units.

## 1. Introduction

Diarrheic diseases in swine have strong economic impacts on production units around the world. In recent years, porcine coronaviruses have represented some of the principal causes of these diseases; however, reports of naturally occurring coinfection of coronaviruses with other viral agents highlight the importance of less characterized viruses on disease severity and outcome [1,2].

Porcine epidemic diarrhea virus (PEDV) induces an enteric disease with high mortality rates in suckling piglets characterized by acute diarrhea and severe dehydration. It was first identified in Europe in 1978 and in Asia in 1982. Since then, PEDV has had a great impact on the Asian swine industry. In May 2013, the presence of PEDV was reported in México along with the United States and Canada, resulting in heavy economic losses for the swine production units [3,4].

PEDV is a member of the *Coronaviridae* family, subfamily *Orthocoronavirinae*, and genus *Alphacoronavirus* and is an enveloped virus approximately 120 nm in diameter with a single-stranded, positive-sense RNA genome of ≈28 kb starting with a 292 nt untranslated region (UTR) divided into six open reading frames (ORFs). The PEDV genome encodes for 16 non-structural proteins (nsp); four structural proteins: spike (S), envelope (E), membrane (M), and nucleocapsid (N); and one accessory protein, ORF3 [5,6].

By contrast, porcine kobuvirus 1 (PKV) is a member of the Kobuvirus genus responsible for gastrointestinal disease, although it has been detected in fecal samples of animals without clinical signs [7]. PKV was discovered in Hungary in 2007 and has been reported in Thailand, China, Japan, Korea, Brazil, the Netherlands, and the United States [7,8,9,10,11,12,13]. This virus is a member of the *Picornaviridae* family, Kobuvirus genus, and Aichivirus C species [14]. Kobuviruses are non-enveloped icosahedral viruses of approximately 30–32 nm. The viral genome consists of 8.2 kb of nucleotides in positive-sense, single-stranded RNA with a poly (A) tail. The genome encodes a viral polyprotein that is divided into the non-structural protein L, three structural capsid proteins (VP0, VP3, and VP1), and seven non-structural proteins [15].

PKV coinfections have been reported for astrovirus, circovirus, rotavirus A, transmissible gastroenteritis virus (TGV), and PEDV [13,16,17,18]. At the same time, PEDV coinfection has also been reported for the same viral agents [2,19,20], including mixed infections of multiple viruses [21]. PEDV and PKV coinfection has been reported in the USA and China, ranging from 15% [8,22] to 81.55% among intestinal samples [23]. 

Another less reported virus in swine is the porcine sapovirus (PSaV), a member of the *Caliciviridae* family. PSaV is a 35-nm non-enveloped virus with a 7.3 kb single-stranded, positive-sense RNA genome. The genome consists of ORF1, encoding the non-structural proteins and the capsid protein (VP1) that is used for genogroup classification, and ORF2, encoding the minor structural protein (VP2) [24]. PSaV has a wide geographical distribution and has been detected in 62% of pigs in the USA with a rate of coinfection with PKV of 10.8% and with PEDV of 13.5% [25,26].

The coinfection of viral diarrheic agents represents a potential complication for disease identification, control, and prevention, particularly in locations with no previous report of these viruses. In this study, we explored next-generation sequencing (NGS) as a tool for the identification of viral pathogens during an outbreak of diarrhea in a commercial farm, making the detection and characterization of natural viral coinfection in diarrheic piglets possible.

## 2. Materials and Methods

### 2.1. Sample Collection

Samples were collected from 10 different farms distributed in four Mexican states, and all tested animals presented clinical manifestations compatible with gastrointestinal disease at the time of sampling. The commercial farrow-to-finisher units tested were located in the states of Nuevo León, Guanajuato, and Estado de México, and samples from the state of Chiapas belonged to backyard swine herds. The production located in Estado de México had a history of previous diarrheic outbreaks caused by PEDV. For the rest of the tested farms, no previous outbreaks caused by PEDV were reported.

A total of 68 rectal swabs from adult swine and six intestinal tissue samples from piglets in litters presenting clinical signs were taken following euthanasia according to the American Veterinary Medical Association (AVMA) Guidelines for the Euthanasia of Animals: 2013 Edition on the farm. The samples were aliquoted and stored at −80 °C in sterile plastic tubes with Dulbecco’s modified Eagle’s medium (DMEM) until processing.

### 2.2. RNA Extraction

RNA extraction of the tissue samples was performed using the RNeasy Mini Kit (Cat no. 74,904 Qiagen, Valencia, CA, USA) according to the manufacturer’s instructions. Approximately 30 mg of tissue samples were disrupted in frozen mortars and Buffer RLT inside a laminar flow cabin. The quantification of extracted RNA was performed with a Qubit RNA HS Assay Kit (Cat no: Q32852 Invitrogen). After extraction, quantification, and quality assessment, the extractions were divided for use in molecular testing and sequencing. 

### 2.3. Molecular Testing

cDNA was synthesized using Superscript VILO (Invitrogen, Hercules, CA, USA) and PCR for PEDV detection was performed using Platinum™ Taq DNA Polymerase High Fidelity (Cat no: 11,304,011 Invitrogen™, Hercules, CA, USA) following the protocol described by Li et al. [27] The reaction was conducted under the following conditions: 94 °C for 2 min, (94 °C for 1 min, 55 °C for 1 min, and 72 °C for 90 min) for 40 cycles, with a final period of 10 min at 72 °C using the following primers targeting the PEDV M gene: 5′-AGTCTTACATGCGAATTGACC-3′ and 5′-AGCTGACAGAAGCCATAAAGT-3′. 

For the detection of PKV, the protocol described by Liu et al. [28] was employed using the primers PK-F10 5′-GGTGGACTCATTGAGTAC-3′ and PK-R10 5′-CCTCCCTGGGTGCAGCTTC-3. The samples were also tested for the presence of Deltacoronavirus using the protocol used by Hu et al. with the primers forward 5′-CGCGTAATCGTGTGATCTATGT-3′ and reverse 5′-CCGGCCTTTGAAGTGGTTAT-3′ [29]. Two PEDV- and PKV-positive intestinal tissue samples of piglets with acute clinical signs were selected for whole genome sequencing and sent to the next-generation sequencing core facility at the Biotechnology Institute, UNAM in cold chain transport.

### 2.4. Sequencing and Bioinformatics Analyses

RNA sequencing of positive samples was performed at the next-generation sequencing core facility at the Biotechnology Institute, UNAM. RNA concentrations were measured (Genova Nano micro-volume spectrophotometer 737,501, Jenway, Staffordshire, UK) and sequencing libraries were constructed using the TruSeq Stranded mRNA Sample Preparation Kit (Illumina, San Diego, CA, USA) using 5 uL of RNA. The samples were then deep sequenced on the Illumina NextSeq500 system, generating paired-end reads of 75 bases. After quality assessments by FASTQC (Babraham Bioinformatics, UK) [30] and adapter elimination using Cutadapt v1.11, the sequence reads were first filtered using Bowtie2 Alignment software against the *Sus scrofa* reference genome (GCF_000003025.6).

The remaining data were assembled with the SPAdes genome assembler v3.12.0 (Center for Algorithmic Biotechnology, St. Petersburg, Russia) and SSPACE v3.0 tool (BaseClear B.V., The Netherlands) [31,32,33]. The assembled contigs were subject to taxonomic classification using the Kraken metagenomic classification pipeline. To identify contigs corresponding to viral genomes, Basic Local Alignment Search Tool (BLASTn) was used against a viral genome database obtained from the NCBI website (available from: https://ftp.ncbi.nlm.nih.gov) (accessed on 20 February 2020) [34,35]. Finally, the original reads were mapped to the viral contigs using the BBMap Alignment software to verify the coverage [25]. Prediction of homologous recombination events was performed using RDP4 (Recombination Detection Program) [26]. The analyses were executed at the Center for Genomic Sciences, UNAM facilities.

### 2.5. Phylogenetic Analysis

Phylogenetic trees were constructed using the maximum likelihood algorithm with SH-like branch support using the PhyML software v3.3.21090321 (Université de Montpellier, France) [36] and iTOL v6.1.1 (University of Würzburg, Germany) [37], with the general time reversible model selected by the sJModelTest v2.1.10 software (University of Vigo, Spain) [38] for all trees.

For the phylogenetic analyses, complete genome sequences of PKV, as well as sequences corresponding to PEDV and the capsid coding region of PSaV, were downloaded from the NCBI database. Then, representative sequences of clusters were selected by CD-HIT-EST [22], and sequences from México were included if available. The representative sequences and obtained genomes were aligned using MAFFT v7.45 (Osaka University, Japan) [39].

For PKV, only the coding sequence (CDS) was used for the phylogenetic reconstruction due to the variability in the lengths of the available sequences. For the PSaV phylogeny, the capsid protein coding region was employed.

### 2.6. Viral Culture

The two original intestinal samples that tested positive for PKV and PEDV by RT-PCR and confirmed by sequencing were propagated separately for virus isolation. Intestinal samples were prepared as 10% homogenates in DMEM and passed through 0.45 µm filters (GE Healthcare, Chicago, IL, USA). The resultant supernatants were inoculated with Vero cells (African green monkey kidney) cultured at 37 °C with 5% CO2 in Dulbecco’s Modified Eagle’s Media (DMEM; GIBCO, Gaithersburg, MD, USA). Cytopathic effects (CPEs) were evaluated on a daily basis and analyzed for viral replication by RT-PCR after every passage.

## 3. Results

### 3.1. Prevalence of Porcine Kobuvirus and Porcine Coronavirus in Diarrheic Pigs

Coinfection of PKV and PEDV was detected in 80% (8/10) of the analyzed farms located in Nuevo León, Guanajuato, and Estado de México states. All the farms presented clinical manifestations at the moment of sample collection. Of the samples used in this study, 51.4% (38/74) were positive for PKV and 67.6% (50/74) were positive for PEDV. Overall, PKV and PEDV coinfection was detected in 36.4% (27/74) of samples. The Deltacoronavirus genome presence was only detected in one farm located in Guanajuato with no PEDV or PKV coinfection.

### 3.2. Viral Genome Detection 

Positive PEDV and PKV intestinal samples belonging to two piglets with acute clinical signs were selected for next-generation sequencing. After the elimination of low-quality reads, an average of 7,029,014 reads per sample were obtained. These reads were assembled, and contigs were taxonomically annotated using Kraken and BLASTn. The Kraken results show the identification of known swine pathogens classified in families *Coronaviridae, Picornaviridae,* and *Caliciviridae*; other viral agents of families *Tospoviridae, Bromoviridae,* and *Marseilleviridae* were also detected. The assembled contigs of swine viruses and their length are shown in Table 1. 

At the species level, sample 205H showed coinfection by PEDV, PKV, and PSaV, while sample 103H showed evidence of PEDV and PKV coinfection, but no presence of PSaV. Two complete PEDV genomes (28,029 and 28,034 nt) were assembled in addition to one full-length sequence corresponding to porcine kobuvirus Aichivirus C (8116 nt), a partial porcine kobuvirus Aichivirus C sequence (580 nt), and a partial porcine sapovirus sequence (2977 nt) (Table 2). BLASTn analyses using an NCBI viral genome database showed high coverage and identity between both PEDV genomes and a USA strain (KF272920.1).

### 3.3. Genome Analysis of Porcine Kobuvirus

**Figure 1:**
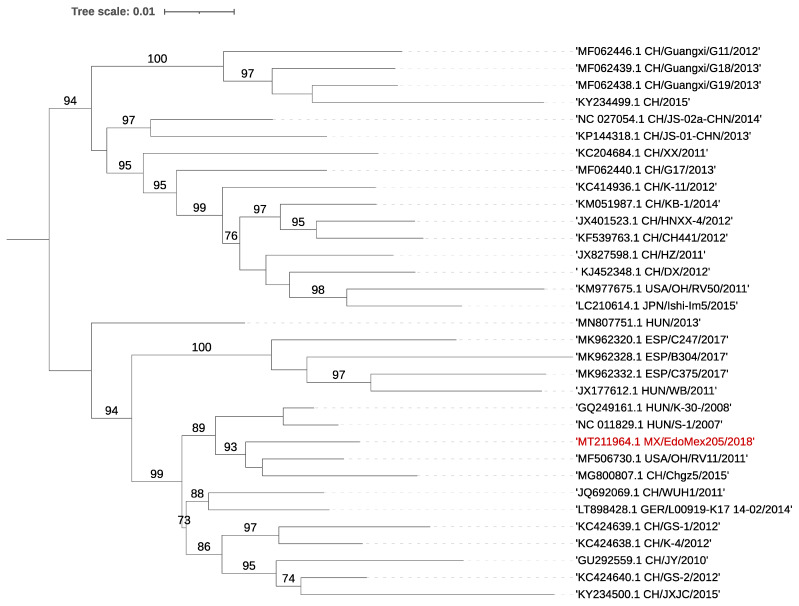
Phylogenetic tree using the full CDS of PKV. The tree was inferred using the maximum likelihood method and GTR model with gamma distribution and SH-like branch support using PhyML software v3.3.21 (Université de Montpellier, France). The sequence obtained in this study is marked in color.

PEDV and Aichivirus C coinfections were identified in the two analyzed samples. A complete sequence of the porcine kobuvirus genome (GenBank Accession no. MT211964) was obtained in sample 205H, and a contig of 10% of the length of the genome was also generated for sample 103H (Table 2). Phylogenetic analysis based on the complete polyprotein gene revealed that the strain detected in this study formed a cluster with isolate OH/RV11/2011 from the USA and Chgz/2015 (Figure 1).

Sequence analyses of the polyprotein gene revealed a nucleotide identity range of 89.70–90.66% and an amino acid identity between 96.91 and 97.84% to reference strains from the USA and China (GenBank: MF506730.1, KC424640.1), respectively. The previously reported 90 nt motif duplex present in strains from Hungary, Spain, and China and the insertion present at position 135–137 nt of US/OH/RV50/2011 were not present in the assembled sequences [14,21]. No evidence of recombination was found.

### 3.4. Genome Analysis of the Porcine Epidemic Diarrhea Virus

**Figure 2:**
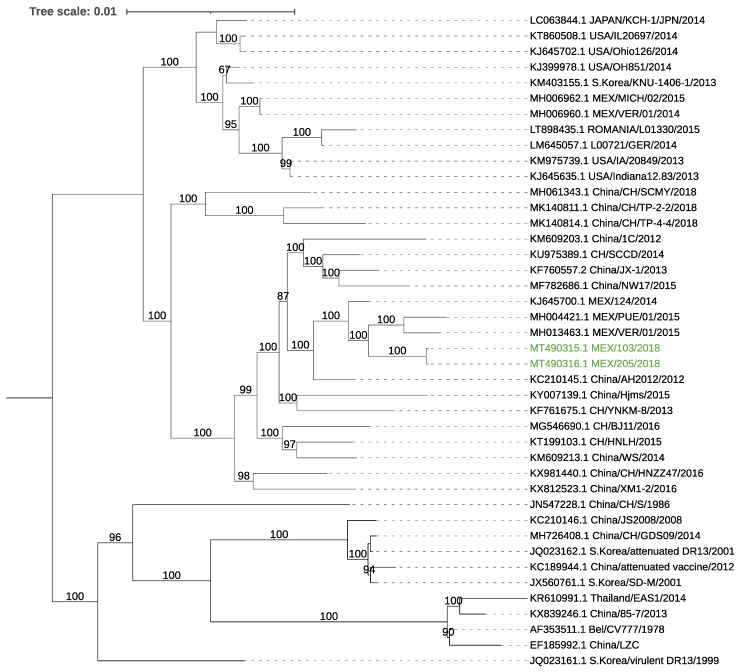
Phylogenetic tree using the full genome sequences of PEDV. The tree was inferred using the maximum likelihood method and GTR model with gamma distribution and SH-like branch support using PhyML software v3.3.21 (Université de Montpellier, France). The sequences obtained in this study are marked in color.

For both samples, full-length coronavirus genomes were assembled. These sequences presented a ≈99.6% identity to local Mexican sequences. Full genome phylogenetic analysis showed that the assembled genomes were located in a cluster with other previously reported no-indel Mexican strains (Figure 2). ORF3 of the two genomes consisted of 224 aa, with a high nucleotide identity (≈99.8%) to local strains, and, as expected from the phylogeny results, did not have the deletion present in attenuated strains [40,41]. However, the two ORF3 sequences showed mutations at the amino acid level: V → A at amino acid position (aa) 21, V → I at aa 54, F → V at aa 80, and A → T at aa 100.

The analysis of the S gene of both samples showed a nucleotide similarity with local strains of 99.47–99.66% and 99.42–99.78% at the amino acid level (MH004420.1, KJ645708.1) and a 99.66% nt/96.64% aa identity to the reference strain CV777 (NC_003436.1), with most amino acid changes located in the S1 domain. In addition, multiple mutations were located among regions containing neutralizing epitopes at residues 592–607: G → S at aa 594, A → E at aa 605, and in the epitope region 636–789: Y → S at aa 766, N → D 707, N → S at aa 719, N → S at aa 724, and Y → S at aa 766 of the reference genome CV777 S protein. The obtained sequences were uploaded to the GenBank database: MT490315 (103H), MT490316 (205H).

### 3.5. Sequence Analysis of PSaV

**Figure 3:**
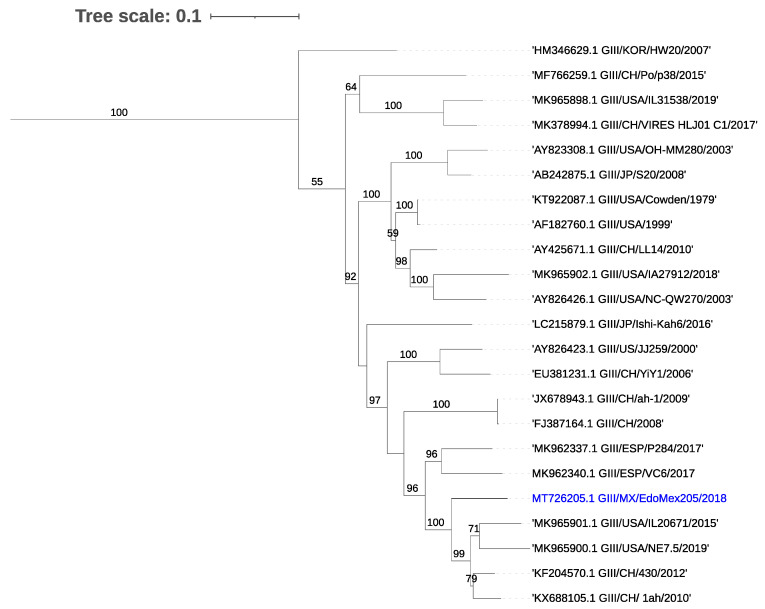
Phylogenetic tree for the capsid coding region of porcine Sapovirus. The tree was inferred using the maximum likelihood method and GTR model with gamma distribution and SH-like branch support using PhyML software v3.3.21 (Université de Montpellier, France). The sequence obtained in this study is marked in color.

A sequence of 2977 nt corresponding to PSaV partial ORF 1 and ORF 2 was assembled from one of the samples, displaying high nucleotide homology (91.13%) to an available Sapovirus GIII sequence (KX688107.1). For VP2, the similarity was 93.36% at the nucleotide level and 97.08% at the amino acid level to the USA 2015 sequence (MK965901.1). The capsid gene presented a homology of 97% at the amino acid level with the GIII 2019 USA strain (MK965900.1). Phylogenetic analysis based on the capsid gene region of PSaV showed a clustering with samples from the USA and China (Figure 3).

### 3.6. Viral Culture

**Figure 4:**
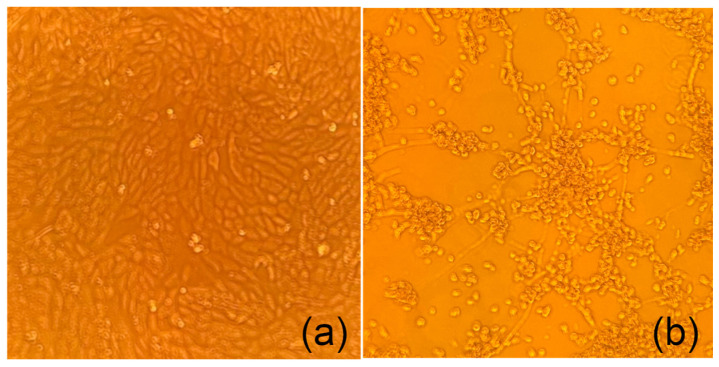
Representative cytopathic effect (CPE) after 5 days post-infection. (**a**) Mock-infected Vero cells. (**b**) Cytopathic effect of Vero cells after passage 9 from infection with gastric content obtained from a diarrheic piglet 10X.

Following inoculation and passage with PKV-positive samples, a cytopathic effect was visible 3 to 4 days post-infection (p.i.). After harvesting, PKV RNA was consistently detected in two isolates after several passages. Porcine coronavirus RNA was not detected by RT-PCR in the cells or supernatants in passages in cell culture, and no cell fusion or syncytia characteristic of porcine coronavirus replication was observed (Figure 4).

## 4. Discussion

Since the introduction of PEDV in México in 2013, it has become one of the primary causative agents for diarrheic manifestations in swine productions. Additionally, PKV was detected in pigs from the majority of analyzed herds. Although PEDV was identified as being responsible for the majority of the outbreaks in the analyzed production units, we were able to identify the presence of PKV in most of these farms. PKV was unknown to be circulating in México and had not been considered in the control measures at the affected farms due to the lack of previous reports in the country.

The presence of both viruses in the same herds with similar clinical signs could indicate a high association of kobuvirus infection with diarrhea but not necessarily as the causative agent since no affected farm was solely positive for PKV. In addition to coronavirus infection by PEDV, diarrheic outbreaks in Mexican porcine farms have been attributed to Deltacoronavirus infection; however, the only farm positive for Deltacoronavirus in this study was negative for coinfection with PEDV and PKV [42].

PKV has been detected in pigs with and without clinical symptoms [43], with the highest prevalence occurring in suckling pigs and 40% positivity in non-diarrheic piglets, as reported by Park, 2010 [13]. It is possible that PKV is an opportunistic pathogen of gastroenteritis in pigs when primary infection by PEDV or other pathogens occurs; however, the possible effects in disease outcome, recovery times, and daily weight gain in surviving animals are unknown. Although the PEDV and PKV coinfection pathogenesis remains unclear, Zhao et al. reported a higher prevalence of this coinfection over the prevalence of infections by PEDV alone in China, which could indicate a possible greater impact and dissemination for coinfection cases [18].

Based on the whole genome phylogenetic analyses, both PEDV and PKV showed a close relation to strains reported in México, China, and the USA. No local sequences for PKV were available at the time of this study. Another agent detected in the NGS analyses was PSaV. The PSaV capsid analysis reflected the same geographical association as PKV and PEDV. The clustering of these viruses reflects the close commercial relation of the swine industry between the USA and México. To our knowledge, this is the first full genome report of porcine kobuvirus in México, as well as the first phylogenetic analysis for porcine PSaV in the country.

The taxonomic classification of de novo assembled contigs by BLASTn reported the presence of several viral agents in the samples, mainly members of Coronaviridae, Picornaviridae, and Caliciviridae families in sample 205H known to cause similar clinical presentations. However, the assembly of full-length genomes for PKV in sample 103H was not possible, and PSaV was not detected. The difference in the detected pathogens may derive from the disparity in the sequencing efficiency, as both animals were housed in close proximity in the same area. Additional analyses based on contigs and filtered reads detected the presence of known commensal and environmental viruses coherent with the type of sample used—likely originating from the digested intestinal content.

During an active outbreak in a production unit, diagnosis is restricted to a small array of agents known to be present in the geographical region; thus, the most prevalent causes such as PEDV are likely to be investigated and controlled, leaving possible secondary infections undiagnosed, which could lead to complications, particularly for feedback-based control. While the implementation of regular viral metagenomics as a regular diagnostic tool could be prohibitive for most farms, an NGS analysis of representative samples from farms could provide a better perspective and understanding of the circulating viruses and other pathogens in the production unit and could be available from a commercial provider. Further studies must be conducted to estimate the prevalence of PKV and PSaV in swine production units and their clinical implications.

## Figures and Tables

**Table 1:** The number of mapped reads and contigs assembled for tissue samples of diarrheic pigs. The swine viral families identified in the analyzed samples.

Sample ID	Number of Filtered Reads	Assembled Contigs	Viral Swine Families	No. of Matched Reads	Scaffold Length (nt)	Mean Coverage
205H	8,606,544.00	360	*Coronaviridae*	309,090	28,029	827.06
			*Picornaviridae*	186,517	8116	1723.60
			*Caliciviridae*	402	2977	10.13
103H	5,451,484.00	333	*Coronavidae*	119,986	28,034	321.00
			*Picornaviridae*	336	580	43.45

**Table 2:** Percentage identities of the assembled contigs for swine viral families.

Sample ID	Assembled Genome (AC Number)	Length (nt)	Subject Cover	Best Hit (BLASTn) (AC Number)	Perc. Identity (AC Number)
205H	EdoMex/205/2018 (MT490316.1)	28,029	99.97%	Porcine epidemic diarrhea virus strain USA/Colorado/2013 (KF272920.1)	99.66% (95.89%)
	EdoMex/2018/205 (MT211964.1)	8116	99.50%	Porcine kobuvirus isolate OH/RV11/2011 (MF506730.1)	90.66% (97.84%)
	EdoMex/2018/205 (MT726205.1)	2977	40.30%	Sapovirus GIII isolate p2 (KX688107.1)	91.13% (96.64%)
103H	EdoMex/103/2018 (MT490315.1)	28,034	99.99%	Porcine epidemic diarrhea virus strain USA/Colorado/2013 (KF272920.1)	99.66% (95.89%)
	NA	580	10%	Porcine kobuvirus strain KobuV/Pig-wt/ESP/P2B/2017 (MK962329.1)	95.86% (100%)

## Data Availability

The data presented in this study are openly available in in the GenBank at https://www.ncbi.nlm.nih.gov/genbank/ (accessed on 20 February 2020), Reference numbers: MT490316.1, MT726205.1, MT211964.1, MT490315.1.
